# Experimental Study on Static and Dynamic Response of Aluminum Honeycomb Sandwich Structures

**DOI:** 10.3390/ma15051793

**Published:** 2022-02-27

**Authors:** Radosław Ciepielewski, Roman Gieleta, Danuta Miedzińska

**Affiliations:** Institute of Mechanics and Computational Engineering, Faculty of Mechanical Engineering, Military University of Technology, Kaliskiego 2 St., 00-908 Warsaw, Poland; radoslaw.ciepielewski@wat.edu.pl (R.C.); roman.gieleta@wat.edu.pl (R.G.)

**Keywords:** aluminum honeycomb, strain rate, Split Hopkinson Pressure Bar, drop hammer, compression

## Abstract

Honeycomb aluminum structures are used in energy-absorbing constructions in military, automotive, aerospace and space industries. Especially, the protection against explosives in military vehicles is very important. The paper deals with the study of selected aluminum honeycomb sandwich materials subjected to static and dynamic compressive loading. The used equipment includes: static strength machine, drop hammer and Split Hopkinson Pressure Bar (SHPB). The results show the influence of applied strain rate on the strength properties, especially Plateau stress, of the tested material. In each of the discussed cases, an increase in the value of plateau stresses in the entire strain range was noted with an increase in the strain rate, with an average of 10 to 19%. This increase is mostly visible in the final phase of structure destruction, and considering the geometrical parameters of the samples, the plateau stress increase was about 0.3 MPa between samples with the smallest and largest cell size for the SHPB test and about 0.15 MPa for the drop hammer test.

## 1. Introduction

The problem of research, design and construction of structures capable of absorbing kinetic energy is a well-known issue that is of interest to scientists and engineers in the automotive, aviation, space, railway and maritime industries. All of the above-mentioned environments struggle with the effects of unexpected dynamic interactions and the accompanying adverse effects affecting the safety of people and equipment. For many years, researchers have focused their efforts on ensuring the highest possible level of protection against the effects of traffic accidents and other random events that are assumed as dynamic loads. In addition to active protection systems, which are to protect against the occurrence of such events, passive protection is equally important, i.e., one that is to protect against the effects of the events or minimize their impact. A special branch of knowledge on human passive safety is the protection of crews of special vehicles, which are used in particular by military forces. It is distinguished primarily by the nature of the interactions, against which protection solutions are designed. Behind the extortion that carries the danger is not an accident or a coincidence, but a planned, deliberate human action.

Increasing trends in the number of fatal attacks with the use of explosives detonated under vehicles and the share of such attacks in all events causing death or permanent injuries of soldiers published at the beginning of the 21st century [1,2] prompted researchers to intensify their work towards finding new, more effective ways to improve the safety of crews, and the state authorities responsible for the army modernization to impose higher requirements regarding mine and ballistic protection. These trends also apply to the Polish Armed Forces, soldiers of which experienced the dangers related to the impact of explosions of improvised explosive devices during operations in foreign missions [3,4].

According to a report [5] published by Action on Armed Violence, which has been keeping statistics on acts of violence continuously since 2010, in the last decade—from October 2010 to the end of September 2020, there were 28,729 incidents involving the use of explosives, as a result of which 357,619 victims were recorded, of which 263,487 were civilians. Of the above-mentioned, 11,971 incidents related to the use of an IED (improvised explosive device). A total of 171,732 people were killed or injured in them, which is 48% of all affected by explosives. Further, 80% of civilians and 20% of soldiers were among all the victims injured by the IED. 

According to reports prepared by American and British agencies [5,6,7], since 2010, mines and IEDs have posed the greatest threat to soldiers of the local armies during hostilities and stabilization operations. Of the 5413 US soldiers killed in an operation where the cause of death was known, approximately 2640 were hit by an IED, and of the 634 British soldiers killed, 273 were attributed to the operation of an IED.

The physical essence and, at the same time, the basic measure of the threat carried by the shock wave resulting from the detonation of an explosive is the value of its positive pressure impulse. Its value is determined from the formula [8]:

(1) $$I^{+}=\int_{t_{a}}^{t_{a}+t^{+}}\left(P\left(t\right)-P_{atm}\right)dt$$

where: $P_{atm}$ is ambient pressure, e.g., atmospheric one, $t_{a}$—time of the shock wave reaching the target, $t^{+}$—duration of the positive wave ($P\left(t\right)>P_{atm}$).

As indicated in the works [8,9], the value of $\max P^{+}$ strongly depends on the distance from the center of the explosive charge—its value can be scaled using the parameter Z described by Hopkinson-Cranz as [8]:

(2) $$Z=\frac{r}{m^{1/3}}$$

where r is a distance from the center of the explosive charge and m is the mass.

The simplest method of counteracting the effects of the shock wave on the vehicle structure is to ensure the maximum distance between the potential hazard location and the vehicle bottom—ground clearance. For obvious reasons, such as limiting the vehicle height, this parameter cannot be freely shaped; therefore, among the methods of minimizing the effects of the impact of the shock wave on the bottom of the vehicle, one of the most popular and effective methods is to reduce the value of the normal component of the pressure acting on the plane of the bottom of the vehicle [10,11]. This is achieved by appropriate shaping of the hull protection elements—most often in the shape of the letter “V”.

At the same time, the requirement to maintain the appropriate ground clearance influencing the off-road capability and the lowest possible mass influencing mobility introduces the necessity to ensure an equally high level of protection for the crews of vehicles equipped with a flat bottom [12].

In the case of this type of structure, the phenomena of kinetic energy conversion into elastic or plastic deformation energy, heat or work are most often used.

There are many ways to use these mechanisms to provide the desired energy absorption capabilities for a given load condition. Thus, they are accompanied by the selection of materials or structures having the desired properties. These are, for example, foamed metals [13] or plastics or structures appropriately shaped from sheet metal or fibrous composites arranged in blocks [14,15]. Among the above-mentioned, there are known structures with an oriented cell structure, including in the form of a honeycomb. They have a long history of successful applications. For example, they were used in the initial period of the conquests of space, in the Apollo project [16], to build elements of the crew and technical compartments. NASA researchers [17], examining a wide range of this type of materials, concluded that the best energy-absorbing properties in relation to the specific mass have elements with a honeycomb structure. Similar conclusions were drawn from studies from the second decade of the 20th century [18,19].

Along with the development of materials with an oriented, cellular structure, their properties began to be used to create combined structures—as a filler for elements, called sandwich structures.

Sandwich structures with honeycomb cores are commonly used because of their high stiffness-to-weight ratio. Moreover, they have predictable progressive failure (stress–strain) characteristics in the case of static loads. This feature made it possible to derive a mathematical model that enables the calculation of the so-called average value of the destructive (plateau) stresses to the core (occurring from the moment of loss of stability to the beginning of the force increase due to the thickening of the structure) on the basis of the known basic values characterizing the honeycomb structures, such as the yield point of the core material, wall thickness and cell size [20]. In the works [21,22], however, the authors show that with dynamic loading, the strength of the structure can increase even up to 50%. In addition, there are a number of aspects, such as air entrapped inside cells [23], the simplification of which may make it impossible to effectively predict structural behavior.

**Figure 1:**
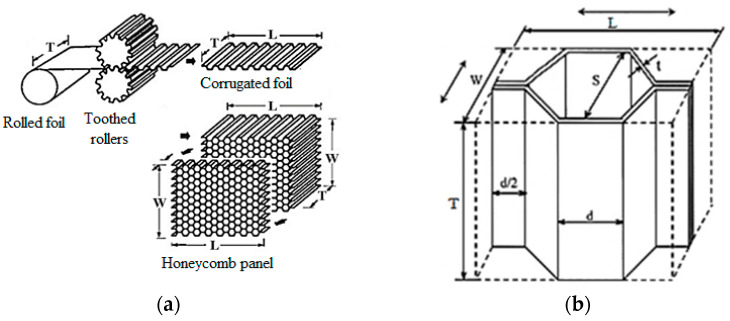
(**a**) Process of aluminum honeycomb development [29]; and (**b**) basic parameters describing structure cell [30].

Honeycomb structures are made of preformed sheets between toothed rolls. The packages of formed sheets are adhesively joined together. The sheets are stacked on top of each other and glued in a way that allows obtaining of repeatable, hexagonal cells. The bundles of pre-shaped sheets are then pressed against each other until the binder solidifies, and then stretched to the desired cell shape. This process is illustrated in Figure 1a.

As a result, a structure is created with characteristic geometric parameters, directly influencing its usability and strength. To clearly define the individual parameters of the structure geometry, appropriate identifiers are assigned. For example, the direction in which consecutive sheet packages are laid and glued, which is also the direction in which there are double walls, is marked with *L*—longitudinal direction. The direction perpendicular to it, in *W*—transverse direction. The height of the core is often marked with *T* or *h* direction. The size of a single cell is marked with the letter *S*, the thickness of the wall—with the letter *t*, and the width of the wall of a single cell—with the letter *d* (Figure 1b).

The methodologies, according to which the experimental research was carried out, are most often drawn up on the basis of American standards [24,25,26,27,28], which strictly define the conditions for carrying out tests determining the parameters of spacers and the cores themselves.

The research methodologies indicated above have been used, also with some modifications, by many researchers dealing with the strength of cellular or porous structures.

The use of all of them to determine the characteristics of a few selected structures is described in [30]. Researchers determined the strength of three types of loaded structures according to standard guidelines. The structures were selected by them in such a way as to assess the influence of the thickness of the film used to make the core and the height of the core. 

A common approach to experimental research is to compress cores without covers. It allows for a significant simplification of the process of preparing test samples and for effective determination of the average destructive force; however, it may be difficult to determine the modulus of elasticity and the maximum critical force that can be transferred by the structure in the elastic range. The work [31] presents the process and results of static tests of honeycomb structures under compression.

Other attempts to hold the structure in place throughout the entire duration of the tests is the method presented in [32], which consists in compressing a sample with dimensions much larger than the moving plane of the testing machine head. 

The choice of the research methodology intended for testing structures without covering plates does not always result from an attempt to simplify the process, but it is also a result of the need to assess the impact on the obtained characteristics of the structure of air entrapped in cells, which is compressed together with the tested core. There are studies that describe approaches to compressing the sample while releasing air from the space between the walls of the core. One of them is described in [23]. The researchers made samples that consisted of a core and two covers with holes drilled in them. The samples were divided into three groups: samples with no holes, samples with holes in 51% of the cells, and samples with holes in 100% of the cells.

Another aspect raised by the researchers is the influence of the strain rate on the average value of stresses destroying the structure. This problem is all the more important as most of the common applications of thin-walled cores of this type are designed to absorb the impact energy or the energy of the shock wave from the detonation of the explosive.

The publication [21] shows that the increase in the average core crushing force under dynamic loads may increase by as much as 50% in relation to that recorded under the static loads. These results were obtained for a deformation velocity of the order of 10^4^ s^−1^ thanks to the use of a compressed air cannon, which was used to drive the samples. The lower influence of the strain rate on the obtained values of the destructive stresses was reported in [33]. 

The most common method of dynamic tests in the strain rate 5 × 10^2^–10^4^ s^−1^ is dynamic compression in the system of elastic bars. The initial assumptions of the Hopkinson method from the beginning of the 20th century [34], was improved by Kolsky 40 years later, which was described in the paper [35] dealing with the study of the mechanical properties of materials deformed at high strain rates. Another modification of the method was made by Lindholm, who described the results of his work in the publication [36] two decades later. At that time, a developed method was called the Split Hopkinson Pressure Bar Test in the scientific nomenclature often referred to as SHPB (Split Hopkinson Pressure Bar). There are known research methodologies using the SHPB method in compression, tensile, torsion and shear tests [37].

In the available literature, there is little information about the conducted research on cell-oriented structures using the SHPB. Two interesting papers from recent years were written by Zhang et al. [38] and Zhao and Gary [39]. The papers present the SHPB tests of selected honeycomb structures, considering their strain rate sensitivity for high strain rates. Further, Hou et al. [40] tested honeycomb structures printed using fused filament fabrication and under shear-compression loading using the modified SHPB method.

The method, however, entails a limitation in the form of dispersion and damping of the elastic wave along the length of the rod. It manifests itself in the gradual distortion and fading of the signal along with the length [41], and thus makes it difficult to interpret the obtained results.

There are algorithms that can reduce this phenomenon. The papers [41,42,43] describe the procedures allowing for the correction of the signals obtained during the tests. This operation enables the prediction of the shape of the elastic wave at the point where the front of the bar affects the sample on the basis of the signal recorded by the strain gauge at a known distance from this front.

**Figure 2:**
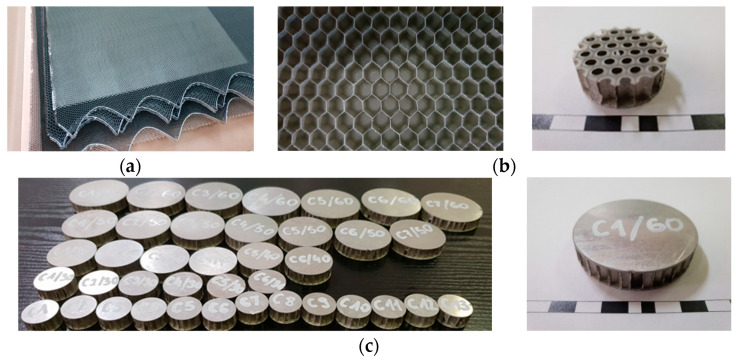
Tested honeycomb material: (**a**) semi-finished products for the production of samples; (**b**) process of joining cores with covers and checking quality of connection; (**c**) samples from series 3/6-5052-.001 prepared for testing.

**Figure 3:**
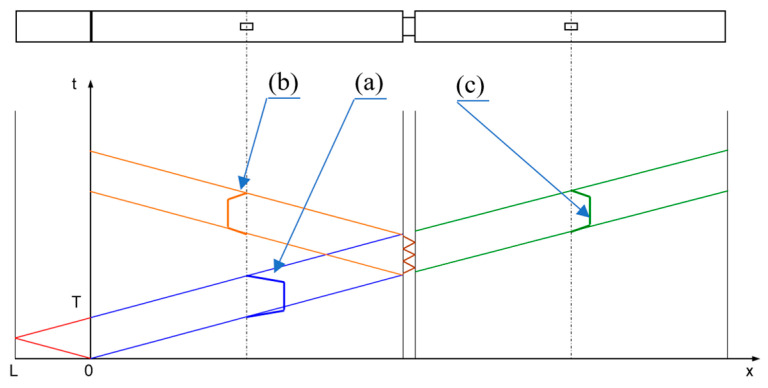
Signals recorded during SHPB tests: (**a**) incident impulse; (**b**) reflected impulse; (**c**) transmitted impulse.

**Figure 4:**
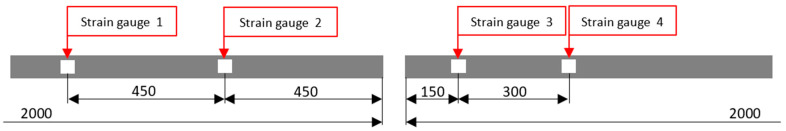
Arrangement of strain gauges on bars.

**Figure 5:**
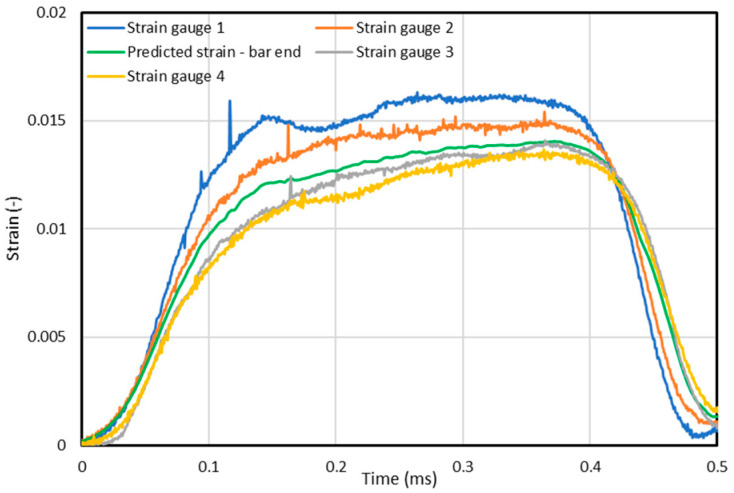
Comparison of signals recorded 900 mm (Strain gauge 1) and 450 mm (Strain gauge 2) from incident bar forehead; signals recorded 150 mm (Strain gauge 3) and 300 mm (Strain gauge 4) from transmission bar forehead and forecast.

In connection with the above literature review, the aim of the article was to conduct static and dynamic tests of selected honeycomb structures and to assess how the load strain rate influences their strength properties. It should be noticed that the complex study of the series of honeycomb structures under a wide range of strain rates and coupling the strength parameters with cell geometry and strain rates (Figure 2, Figure 3, Figure 4 and Figure 5) is not presented in the world literature and can be considered as innovative input in the knowledge of protective panels design and properties.

## 2. Materials

Seven types of structures meeting the test requirements, available in the catalogs of manufacturers of this type of honeycomb plates, were selected. The geometrical parameters of the individual structures are presented in Table 1. The names of each structure type include the following information: cell_size-aluminum_type -wall_thickness, all dimensions are in inches.

As can be seen, the types of structures were selected in a way that allows for the subsequent comparison of the results obtained in the tests by changing only one of the parameters. It is possible to distinguish one group of samples with the same wall thickness *t* = 0.0254 mm and two groups of samples with the same cell size *S* = 3.175 mm and *S* = 4.7625 mm. The ready-made honeycomb core plates intended for the production of test specimens are shown in Figure 2a.

The aluminum honeycomb panels (CR-PAA™, HexWeb^®^, Hexcel Corporation, Stamford, CT, USA) were adhesively bonded with the epoxy resin (EPIDIAN^®^ 53, Ciech Szarzyna S.A., Nowa Szarzyna, Poland) with the hardener (Z-1, Ciech Resins, Szczecin, Poland ) mixed in the proportion of 10:1. Aluminum plates with dimensions of 500 mm × 500 mm × 0.5 mm were used as covers. 50 g ± 3 g of binder was used for each cover, which, assuming that the density of the epoxy resin is 1.19 kg/dm^3^, gives an average thickness of the adhesive layer of 1.6mm. In order to check the level of wetting of the structure walls with the adhesive, one of the covers was removed from the selected samples. The view of the one-sidedly glued core and samples after removing the cover is shown in Figure 2b.

The next stage of preparation for testing is cutting circular samples with a diameter of 25 mm, 30 mm, 40 mm, 50 mm and 60 mm. The size of the sample was varied in order to experimentally and statically investigate the influence of the shore on the obtained results. The samples were made using a water jet cutter (Streamcut 4121, Kimla, Częstochowa, Poland), then the flat surfaces of the samples were ground using a belt grinder in order to eliminate the possibility of contact with the surfaces of the testing devices other than the entire plane of the cover. The samples from the series 3/16-5052-.001 made are shown in Figure 2c.

## 3. Research Methods

The research methods were selected from among those listed in Table 2.

Methods selected for the research allow the determination of the characteristics of the material in the quasi-static and dynamic range of low and medium strain rates without the need to accelerate the sample. This criterion was introduced due to the relatively low stiffness of the core and the high risk of loss of stability of its walls in front of the actual part of the test, which would make it worthless.

### 3.1. Quasi-Static Testing

The static tests were performed with the use of the INSTRON 8802 universal testing machine (Norwood, MA, USA). The tests were carried out at a constant vertical velocity of the head of 5 mm/min in the range of 0–8mm. During the tests, the relative head, the force of reaction and the image were recorded with the PHANTOM V12 high-speed camera (Tokyo, Japan). Using this method, the structure stiffness measurement was carried out with the use of samples with diameters in the range of 25–60 mm.

### 3.2. Dynamic Low Strain Rate Testing

Dynamic uniaxial compression of the structure was carried out using a drop hammer. It is based on the use of the hammer’s kinetic energy to deform the sample placed on the table situated directly under it.

The tests were carried out at the initial vertical velocity of the hammer of ~3.7 m/s. During the tests, the relative hammer displacement, the reactions force was measured and the image was recorded using the PHANTOM V12 high-speed camera. 

### 3.3. Dynamic Mid Strain Rate Testing

The SHPB (Split Hopkinson Pressure Bar) test stand is a system of three elastic bars placed coaxially in longitudinally bearing supports. The first of the bars—the striker is placed in the barrel of a launcher powered by compressed gas. The other two, most often of the same length and diameter, are used to indirectly measure the value of stresses and strains in the tested material over time. This ability is provided by strain gauges adhesively attached to their surface. The test sample is placed between the incident and transmission bars. A striker fired from the barrel hits the incident bar causing the formation of a mechanical elastic wave moving from its forehead to its end. As a result, at the end of the bar, its elastic elongation occurs, and at the same time, the sample is deformed. The part of the elastic wave not absorbed by the deformed sample is transmitted to the transmission bar, through which it also moves from the front to the end. 

The first of the presented measuring blocks is a device for recording the velocity of the striker immediately before the moment of hitting the incident bar. The velocity value in the experiment preparation process can be determined from the formula:

(3) $$v_{x}=\sqrt{\frac{2\cdot P_{0}\cdot V_{0}}{m_{p}}ln\left[\frac{V_{0}+A_{p}\cdot x}{V_{0}}\right]}\;$$

where $v_{x}$ is a striker velocity (m/s); $P_{0}$—initial pressure in a vessel (Pa); $V_{0}$—pressure vessel volume; $m_{p}$—striker mass (kg); $A_{p}$—striker guide hole area (m^2^); x—coordinate of a striker position in a guide.

Calculating the velocity of the striker can be used to describe the stress value in the incident bar from the relationship:

(4) $$\sigma_{I}=\frac{1}{2}\rho_{I}v_{I}v_{x}\;$$

where $\sigma_{I}$—stress in the incident bar (Pa);$\;\rho_{I}$—density of the incident bar material (kg/m^3^); $v_{I}$—velocity of the elastic wave propagation in the incident bar (m/s); $v_{x}$—striker impact velocity on the incident bar (m/s).

The length of the elastic wave pulse passing through the bars is mainly influenced by the length of the striker and the value of the velocity of the propagating wave in the material of the striker. It can be estimated according to the formula:

(5) $$t=\frac{2a_{s}}{v_{p}}\;$$

where t—time of impulse duration (s); $a_{s}$—striker length (m); $v_{p}$—velocity of propagation of an elastic wave in the striker.

From the moment of the shot, three significant courses of deformation values in the bars are recorded. All of them are schematically shown in Figure 3. The first color marked in blue is the incident impulse, which is the information about the requirement affecting the tested sample. The second one—marked in green—is a transmitted wave impulse. It is a residual wave, created as a result of partial attenuation of the incident wave by deforming the sample between the bars. Its course results directly from the value of the reaction force acting on the front of the transmission bar. This means that, using the generalized Hook’s law, this signal can be used to determine the value of the compressive stresses in the sample over time. The last of the recorded waveforms is the reflected wave. It is the incident wave disturbed by the deformation process and is used to determine the value of deformation and the rate of deformation.

The courses of individual values in time are determined according to the following relationships [45]:

- Stress:

(6) $$\sigma \left(t\right)=\frac{EA_{po}}{A_{pr}}\varepsilon_{T}\left(t\right)\;$$

- Strain:

(7) $$\varepsilon \left(t\right)=-2\frac{v}{a_{p}}\overset{t}{\underset{0}{\int }}\varepsilon_{R}\left(t\right)\mathrm{dt}$$

- Strain rate:

(8) $$\dot{\varepsilon }\left(t\right)=-2\frac{v_{0}}{a_{p}}\varepsilon_{R}\left(t\right)\;$$

where $v_{0}$ is a velocity of propagation of the elastic wave in the incident bar; $a_{p}$—sample length; E—Young’s modulus of the bar material; $A_{po}$—cross-sectional area of the transmission bar; $A_{pr}$—sample cross-sectional area; $\varepsilon_{R}$—value of strain in time in the incident bar—reflected wave; $\varepsilon_{T}$—value of strain in time in the transmission bar—transmitted wave.

The SHPB stand was adapted to the use of bars with a diameter of 25 mm. The two bars used were 2000 mm in length. A striker with a length of 250 mm was used. 

To measure the deformation value, strain gauges connected in a quarter-bridge system were used. The signals were amplified using the LTT 500 fast waveform amplifier from Labortechnik Tasler GmbH (Würzburg, Germany). Waveforms were recorded using National Instruments NI USB-6366 high-speed A/D card (Austin, Teksas).

As the structure under study is characterized by low stiffness compared to the materials that are most often used for bars for SHPB tests, i.e., steel or aluminum, the signals obtained were characterized by strong noise. The values of deformation of the transmission bar over time turned out to be too low to be suitable for the analysis. There are several ways to reduce the stiffness difference between the test material and the bars. These include increasing the sample diameter, using hollow bars and using bars made of materials of lower stiffness [39,45,46]. Each of the above-mentioned methods has certain limitations that complicate the correct interpretation of the results. While in the case of the most commonly used metal bars, where the damping or the phenomenon of dispersion are negligible from the test point of view, the recorded signal at a certain distance from the bar face in contact with the sample can be directly used to analyze the properties of the tested material without affecting the quality of the results obtained, the use of polymer bars makes it necessary to apply the extrapolation procedure in order to obtain the correct wave parameters at the bar ends.

In order to identify the phenomena described above and to enable the elimination of their effects, a test was carried out according to the assumptions of the study in the elastic bar system (SHPB), but without placing a sample between them. On both bars, two strain gauges were placed according to the diagram in Figure 4. Due to the fact that the incident bar is used to record both the incident and reflected wave, it was decided to move the last strain gauge, marked with the number 2–450 mm away from the forehead of the rod. This prevents the risk of interference between the beginning of the reflected wave and the end of the incident wave. Such a risk does not occur in the case of the wave recorded on the transmission bar; therefore, the movement of the strain gauge from the front of the bar was intended only to minimize the risk of damaging it.

The result of the test is shown in Figure 5. The differences between the signals recorded on individual bar lengths are clearly noticeable. These are mainly a decrease in the amplitude of the wave and an increase in the period. It is not possible to extrapolate the signal to the point at the end of the bar directly with the use of the scaling factor. To achieve this goal, a broader analysis is necessary.

The successfully applied method for signal extrapolation [41,42,43,44] is based on spectral analysis based on the Fast Fourier Transform (*FFT*). It consists in determining the damping coefficient and the wavenumber, i.e., the inverse of the wavelength of the spatial frequency, which tells about the number of sinusoidal components per unit length. Then, a reconstruction of the frequency spectrum of the signal is performed at the appropriate location on the bar.

Two different points were located on the bar at the distances $x_{1}$ and $x_{2}$, and the time histories of $\varepsilon_{1}\left(t\right)$ and $\varepsilon_{2}\left(t\right)$ were recorded there, respectively. For signals recorded digitally with a constant sampling rate, where the sample number will be marked as τ, the spectra calculated using the *FFT* will have the following form [41]:

(9) $$Y_{1}\left[\omega \right]=FFT\left\{\varepsilon_{1}\left[\tau \right]\right\}=\underset{n}{\sum }Y_{1n}e^{i\phi_{1n}}\;$$

(10) $$Y_{2}\left[\omega \right]=FFT\left\{\varepsilon_{2}\left[\tau \right]\right\}=\underset{n}{\sum }Y_{2n}e^{i\phi_{2n}}\;$$

where $Y_{1n}$ and $Y_{2n}$ are amplitudes, $\phi_{1n}$ and $\phi_{2n}$ represent the phases of the spectral components.

The attenuation coefficient ${\hat{\alpha }}_{n}$ and the wavenumber ${\hat{k}}_{n}$ are parameters that can be estimated on the basis of the digital representation of the measured quantities using the relationship [41]:

(11) $${\hat{\alpha }}_{n}=\frac{lnY_{1n}-lnY_{2n}}{x_{2}-x_{1}}\;$$

and

(12) $${\hat{k}}_{n}=\frac{\phi_{1n}-\phi_{2n}}{x_{2}-x_{1}}\;$$

If the distance from the bar front, in which the time course of the deformation value is to be estimated, is marked as x, then according to the theory of spectral analysis of waves [41], for one-dimensional, linear propagation, the transition function in the bar (transfer function) can be expressed as:

(13) $$G\left[\omega \right]=\underset{n}{\sum }G_{n}=\underset{n}{\sum }e^{-\left(\alpha_{n}+i{\hat{k}}_{n}\right)x}$$

Therefore, if the value of strain in time $\varepsilon_{m}\left(t\right)$ is measured along the length $x_{m}$ of the bar, then along the length $x_{c}$ of the bar on which the course $\varepsilon_{c}\left(\tau \right)$ is to be estimated, its frequency spectrum ${Ε}_{c}\left[\omega \right]$ can be calculated using the following equation [40]:

(14) $$Y_{c}\left[\omega \right]=\underset{n}{\sum }Y_{mn}G_{n}e^{i\phi_{mn}}=\underset{n}{\sum }Y_{mn}e^{i\phi_{mn}}e^{-\left(\alpha_{n}+i\hat{k}\right)𝛥x_{c}}\;$$

where

(15) $${𝛥}_{x_{c}}=x_{c}-x_{m}\;$$

while $Y_{\mathrm{mn}}e^{i\phi_{\mathrm{mn}}}$ is obtained from the *FFT* analysis of the course $\varepsilon_{m}\left(t\right)$, obtained for the length $x_{m}$. Finally, the discrete form $\varepsilon_{c}\left[\tau \right]$ of the $\varepsilon_{c}\left(t\right)$ waveform can be computed using the Inverse Fast Fourier Transform [41]:

(16) $$\varepsilon_{c}\left[\tau \right]=IFFT\left\{Y_{c}\left[\omega \right]\right\}\;$$

The above-presented methodology of predicting and reconstructing the signal in a different place on the bar than in the location of strain gauges is based on the thesis that, on the basis of two known signals registered in different places of the same rod, along which a mechanical longitudinal wave moves free of other disturbances, it is possible to define its shape in any other place located on this rod. For this operation to be effective, the analysis should not subject the signal in a simple form—as a function of time, but the intensity distribution of their harmonic components as a function of frequency—spectrum. After obtaining the parameters determining the signal change (attenuation) and building on their basis, known from transmittance control systems, it is possible to make a forecast of the signal value course in time, after earlier carrying out the inversion of the Fourier transform. The effect of the discussed action is shown in Figure 5 (green curve).

**Figure 6:**
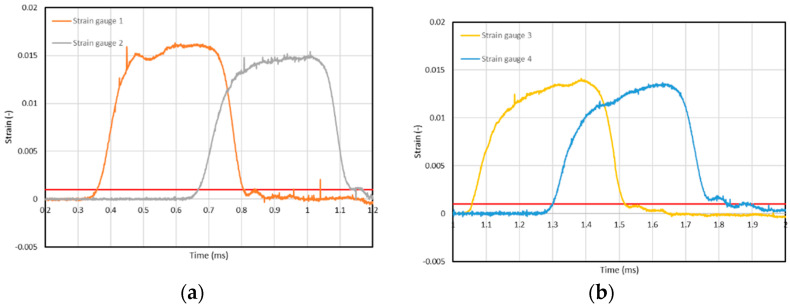
Values of strain vs. time recorded by strain gauges placed on the following bars: (**a**) incident; (**b**) transmission.

Another element necessary to determine the properties of the stand is the measurement of the velocity of the mechanical wave moving along the bar. Taking advantage of the fact that each of them was equipped with two strain gauges, all of them were used to make the measurements. Figure 6 shows the changes of the recorded deformation values as a function of time in the bars under the test during which the bar fronts were associated with each other. In order to establish the wave velocity, the time during which the deformation value at individual points was 0.1% was read (marked with the red line).

**Figure 7:**
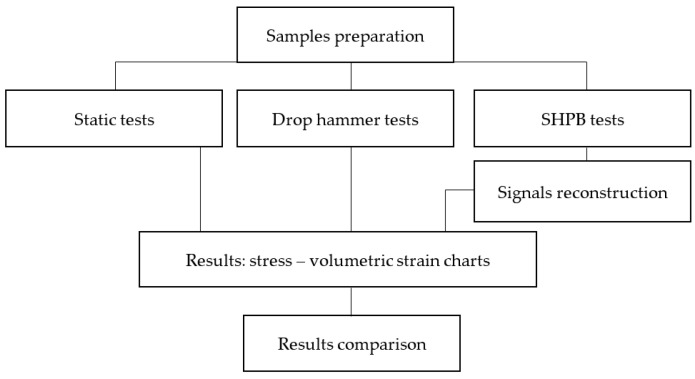
Schematic diagram of experimental research process.

The schematic diagram of the experimental research process is presented in Figure 7.

## 4. Results 

The results of all the tests carried out were collected and analyzed in terms of the correctness of the test and possible non-compliance. In order to eliminate gross errors, the three-sigma rule was applied; therefore, any test result outside the acceptable range of the mean value ± three standard deviations was rejected and the test was repeated. With the normal distribution of the results obtained in this type of research, 99.7% of the observations should be within the acceptable range.

**Figure 8:**
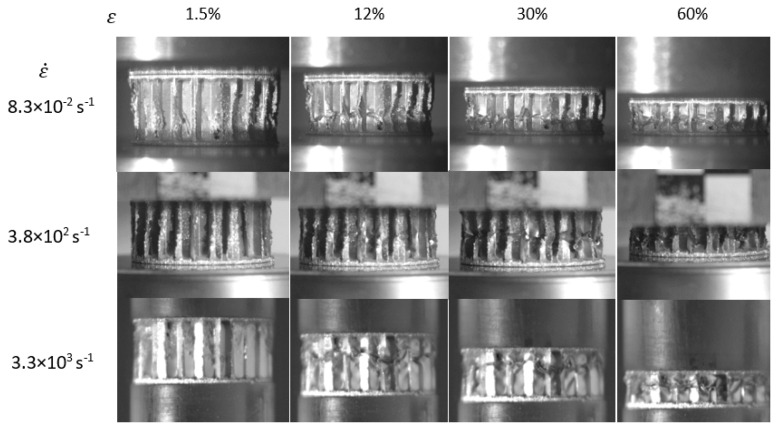
Frames recorded during series 1/8-5052-.0015 tests at different strain rates.

First, the focus was on the analysis of the images captured by the high-speed camera. The recorded images show that the cellular core destruction method for all applied strain rates is similar across the entire strain range. After the first local loss of stability, the first folds are built on the walls. After the fold is closed, the ends of the arch forming it comes into contact, another local loss of stability occurs. After all the folds are formed and closed, the structure is densified by bending the arches formed on the walls. A noticeable difference, albeit not observed in every case, was the shifting of the loss of stability zone and the first wave towards the load along with the strain rate increase. The selected recorded frames arranged according to the strain rate in individual deformation phases for the series 1/8-5052-.0015 are shown in Figure 8.

The next stage of processing the results obtained in the course of the research was the preparation of comparative graphs containing the stress value waveforms related to the volumetric strain values. In each of the graphs presented below, the average curve obtained by performing static compression of five samples is marked in blue—in the legend of the graph marked as QS (quasi-static), the average curve obtained in dynamic low strain rate tests on the drop hammer stand is marked in red—in the legend of the chart marked as DH (drop hammer), while in orange—the averaged result of dynamic mid strain rate tests of five samples on the split Hopkinson bar—in the legend of the chart marked as SHPB (split Hopkinson pressure bar).

**Figure 9:**
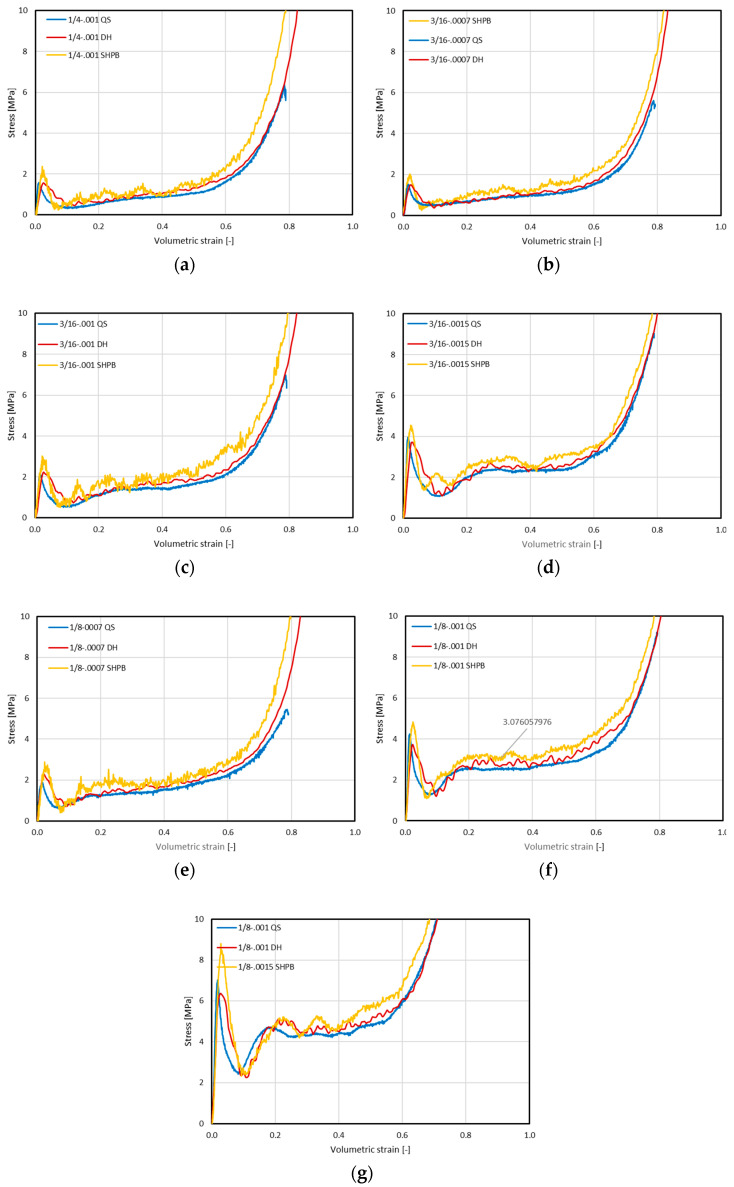
Set of stress vs. volumetric strain characteristics for samples series: (**a**) 1/4-5052-.001; (**b**) 3/16-5052-.0007; (**c**) 3/16-5052-.001; (**d**) 3/16-5052-.0015; (**e**) 1/8-5052-.0007; (**f**) 1/8-5052-.001; (**g**) 1/8-5052-.0015 for tested strain rates (QS—quasi-static, DH—drop hammer, SHPB—Split Hopkinson Pressure Bar).

Figure 9a shows the results of tests carried out on the 1/4-5052-.001 structure. There is a noticeable difference in the stress values in the entire range of volumetric strains, especially when comparing the results obtained in static tests and SHPB tests. The difference in characteristics in the elastic range is also clearly visible.

The value of the crushing stresses described by the determination of the dominant for the entire range of volumetric strain was: 0.906MPa for the static test, 1.011 MPa for the dynamic test with a drop hammer and 1.192 MPa for the SHPB test.

The results obtained from the remaining series of tests were presented in Figure 9b–g. In each case, data from the same series—the same topology of the studied structure—were confronted with each other. It can be noticed that in each of the discussed cases, an increase in the value of stresses in the entire deformation range was noted with an increase in the strain rate. This increase is most visible in the final phase of structure destruction, just before the phase of complete dense (full compression).

The increase in the stress value, associated with the beginning of the core full compression phase—the gradual elimination of voids inside the cells, took place the earlier, the higher the strain rate was used in the test. In the case of the structure 3/16-5052-.0007, the beginning of this phase recorded in the SHPB test stand was already noted at the volumetric strain of 0.41, and during the static test—for 0.55 strain.

There is also a visible tendency for an earlier increase in the failure stress (earlier densification of the structure), and thus a shortening of the range of average crushing stress (plateau), along with an increase in the thickness of the core wall. It is a phenomenon resulting from the arrangement of successive folds formed on the walls along the height of the core. The earlier start of the compaction phase is caused by the presence of folds with a larger radius and their greater stiffness. Their complete closing causes an increase in the reaction force, because the fully compressed structure has a stiffness similar to that of the material from which it is made—in the examined case, it is an aluminum alloy. The greater wall thickness, after closing the folds, will create a greater height of the rigid material, thus reducing the share of the height at which the core is still slack.

## 5. Discussion

On the basis of a simple summary of the obtained results of static tests, it was found that with an increase in the volume of the material (aluminum alloy) in the volume of the sample/core, i.e., with an increase in wall thickness, or a decrease in cell size, the value of critical stresses increases, which leads to a loss of stability and the mean plateau stress. There is also a noticeable influence of the cell size on the shape of the characteristic just after the loss of stability, before reaching the stabilization of the mean failure stress. The first wave shaping phase then occurs, during which a significant decrease in stress is recorded—from 44% for the 1/8-.0015 structure to 63% for the 3/16-.001 structure without a clear trend. The influence of the cell size is visible in the width of the volumetric strain interval during which the first fold shaping phase takes place. In the case of cores with a cell size of 3.175 mm (1/8″), the next extreme after the critical stress occurs when the volumetric strain value is from 0.069 to 0.085, in the case of a cell size of 4.7625 mm (3/16″) volumetric strain is from 0.089 up to 0.118, and for a cell size of 6.35 mm (1/4″) it is 0.128. The analysis of each series showed that the value of volumetric strain, in which the extreme is recorded, increases with the increase in the core wall thickness.

It should also be noted that structures with a wall thickness of 0.0381 mm (0.0015″) earlier than the others entered the phase of densification (increase in the stress value) and total compaction (final transition to an elastic characteristic).

From the obtained stress vs. volumetric strain characteristics, the average failure stresses were determined by the dominant among the recorded stress values from the entire range of volumetric strains. In Table 3, the basic parameters of all examined structures are listed.

**Figure 10:**
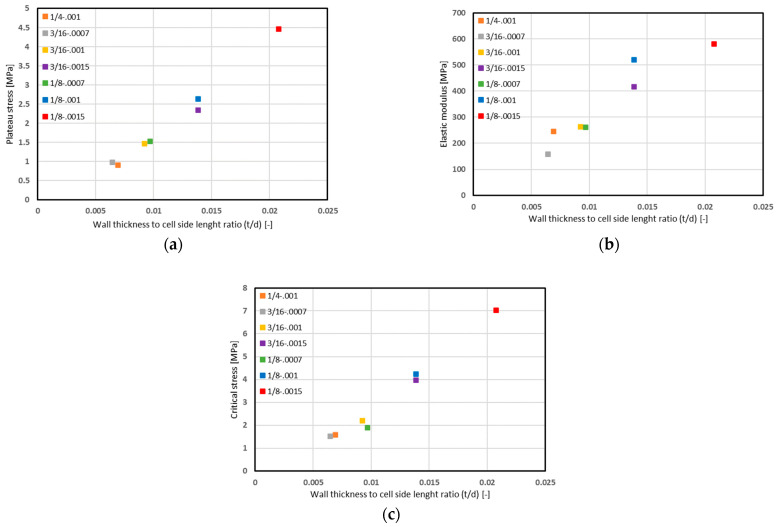
Relationship between (**a**) plateau stress value, (**b**) modulus of elasticity, (**c**) critical stress and ratio of wall thickness to the single core cell side length.

As can be seen, similar to the recorded values of the failure stress, the modulus of elasticity and the values of the critical stress increase with the increase in the wall thickness, while it decreases with the increase in the cell size. Parameters to which selected mechanical properties of porous or cellular materials can be referred are their density (mass to volume ratio) or the ratio of wall thickness to cell side length (*t/d*). Such combinations are presented in Figure 10. The linear relationship between the value of the plateau stress, the modulus of longitudinal elasticity, the critical stress to the dimension ratio *t/d* is clearly visible. Nevertheless, in the case of structures with the same wall thickness to cell size ratio (3/16-.0015, 1/8-.001), a higher value for all the parameters mentioned was recorded for a core with a smaller cell size.

Subsequently, the obtained values of the plateau stress for the individual structure types obtained for the three strain rates are summarized. In each of the tested cases, an increase in the value of the plateau stress was noted with an increase in the strain rate. Detailed results are presented in Table 4.

**Figure 11:**
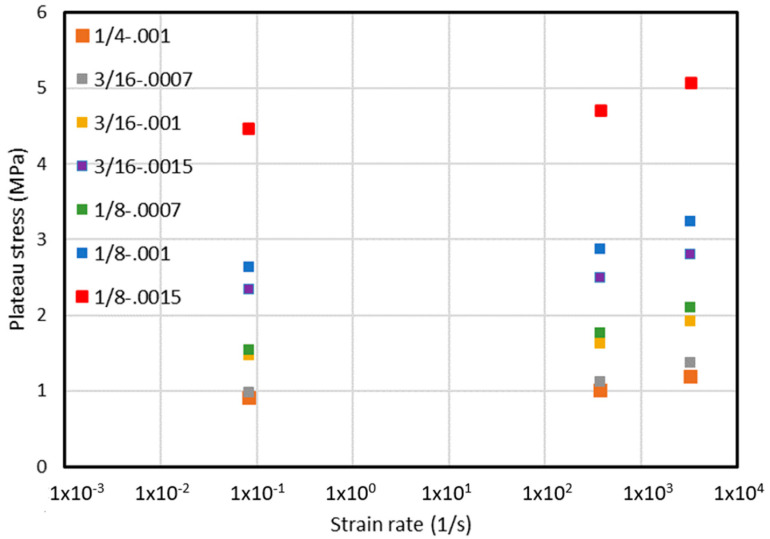
Values of plateau stress depending on strain rate.

In the next step, the obtained average values of failure stresses were placed on a graph, the ordinate of which was given a logarithmic scale on the basis of 10, representing the value of the strain rate. It is presented in Figure 11.

**Figure 12:**
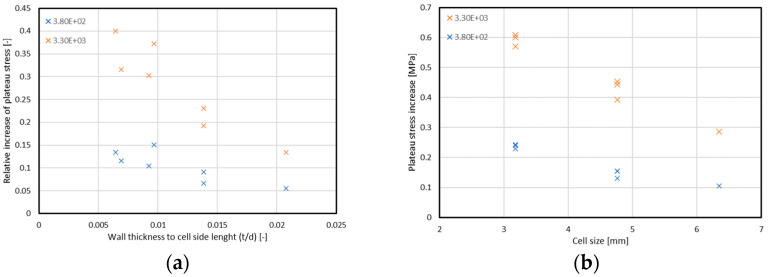
Relationship between value of relative increase in plateau stress and (**a**) ratio of wall thickness to the single core cell height, (**b**) core cell size.

In connection with the identification of the relationship between the individual strength parameters of the cores and the ratio of the wall thickness to the cell size, the relative increases in the plateau stress value obtained at the strain of 3.8 × 10^2^ and 3.3 × 10^3^ 1/s. The chart showing the summary is shown in Figure 12. The increase in plateau stress was not higher with an increase in wall thickness to cell side length ratio. This may prove that there is no relation between the share of material (aluminum) in the core volume and plateau stress increasing. This fact confirms the insensitivity of the material used for the construction of the core (Al5052) to the strain rate, or, as can be found in the literature [47], the material strength decreases along with the strain rate increase. At the same time, it leads to the conclusion that the reason for the increase in the mean failure stress should be seen in the aspects not related to the core material.

Surprisingly, the relationship between the increase in plateau stress and the geometric parameter of the structure was observed in relation to the size of the cell. Structure types with the same cell size showed very similar values of the stress increment; however, with the increase in the cell dimension, this increment was smaller and smaller. The above conclusion was based on the characteristics presented in Figure 10b. It should be noticed that the sample area is limited, so cell size decrease means an increase in the area fully closed by the core walls and in connection—an increase in air volume entrapped inside.

## 6. Conclusions

The complex study of a commercially available series of aluminum honeycomb structures loaded in static, dynamic and SHPB tests was presented. The coupling between geometric parameters of cores and strain rates with plateau stress was examined. This aspect of the honeycomb testing is novel and can be applied in, e.g., protective honeycomb panels design process.

On the basis of the achieved identification of the above-described relationship, it should be assumed that the increase in the value of the plateau stress is related to the air volume entrapped inside the closed cells. Researchers looking for the reasons for the increase in compressive strength of aluminum foams with closed pores came to similar conclusions [48,49], in contrast to those who tested open-cell foams [50]. These premises state that the phenomena related to the compression of the air inside the core are the main, if not the only source of plateau stress increase during dynamic compression. The change of the value of this increment with the change of the cell size may be related to the fact of the limited, but the same volume of the tested samples and the variable volume of the air core closed with walls.

## Figures and Tables

**Table 1:** Geometric parameters of structures intended for research.

No.	Series	Cell Size *S* (mm)	Cell Thickness *t* (mm)	Height *T* (mm)
1	1/8-5052-.0007	3.1750	0.01778	10
2	1/8-5052-.001	3.1750	0.02540	10
3	1/8-5052-.0015	3.1750	0.03810	10
4	3/16-5052-.0007	4.7625	0.01778	10
5	3/16-5052-.001	4.7625	0.02540	10
6	3/16-5052-.0015	4.7625	0.03810	10
7	1/4-5052-.001	6.3500	0.02540	10

**Table 2:** Summary of research methods with the achieved strain rates [44].

Strain Rate [s^−1^]	Scope of Research	Research Methods and Equipment
10^−9^–10^−8^	Creep, stress relaxation	Creepers Hydraulic testing machines
10^−7^–10^−2^	Quasi-static	Hydraulic, servo-hydraulic, screw strength machines
10^−1^–10	Dynamic low strain rate	Hydraulic, pneumatic testing machines Drop hammers, slide hammers Cam machines
10^2^–10^4^	Dynamic mid strain rate	Split Hopkinson Pressure Bar Taylor test Ring test
10^5^–10^7^	Dynamic mid high rate	Shock wave testPlate-to-plate test

**Table 3:** List of basic parameters of the examined structures.

Sample Series	Plateau Stress (MPa)	Elastic Modulus (MPa)	Critical Stress (MPa)	Relative Volume for Fully Dense Material (-)	Elastic Modulus For Fully Dense Material (MPa)
1/4-.001	0.91	245.32	1.57	0.31	36.75
3/16-.0007	0.98	157.64	1.51	0.26	41.74
3/16-.001	1.47	262.85	2.19	0.27	44.26
3/16-.0015	2.34	416.75	3.96	0.29	51.74
1/8-.0007	1.53	261.20	1.90	0.25	28.99
1/8-.001	2.64	519.82	4.24	0.26	52.22
1/8-.0015	4.47	580.00	7.03	0.30	80.05

**Table 4:** Summary of plateau stress values obtained during compression of all types of structures at three strain rates.

Sample Series	Strain Rate (1/s)		
	8.3 × 10^−2^	3.8 × 10^2^	3.3 × 10^3^
	Plateau Stress (MPa)		
1/4-.001	0.906	1.011	1.192
3/16-.0007	0.980	1.111	1.372
3/16-.001	1.469	1.623	1.913
3/16-.0015	2.344	2.499	2.796
1/8-.0007	1.531	1.761	2.101
1/8-.001	2.635	2.875	3.243
1/8-.0015	4.466	4.710	5.066

## Data Availability

Data available after contact with authors, not publicly archived.
